# Multiple correspondence analysis as a tool for examining Nobel Prize data from 1901 to 2018

**DOI:** 10.1371/journal.pone.0265929

**Published:** 2022-04-01

**Authors:** T. Alhuzali, E. J. Beh, E. Stojanovski

**Affiliations:** School of Information & Physical Sciences, College of Engineering, Science and Environment, University of Newcastle, Callaghan NSW, Australia; University of Florida, UNITED STATES

## Abstract

The main goal of this paper is to examine Nobel Prize data by studying the association among the laureate’s country of birth or residence, discipline, time period in which the Nobel Prize was awarded, and gender of the recipient. Multiple correspondence analysis is used as a tool to examine the association between these four categorical variables by cross classifying them in the form of a four-way contingency table. The data that we examine comprise Nobel Prize recipients from 1901 to 2018 (inclusive) from eight-developed countries, with a total sample of 785 Nobel Prize recipients. The countries include Canada, France, Germany, Italy, Japan, Russia, the British Isles, and the USA and the disciplines in which the individuals were awarded the prizes include chemistry, physics, physiology or medicine, literature, economics, and peace.

## Introduction

The Nobel Prize is a prestigious international award that was created in 1901 and awarded to individuals who make significant contributions to the fundamental understanding in the disciplines of physics, chemistry, physiology or medicine, literature, economics, and peace. The Royal Swedish Academy of Sciences awards the Nobel Prize in the disciplines of physics and chemistry, while the Nobel Prizes in physiology and medicine are awarded by the Nobel Assembly at the Karolinska Institute in Sweden, and the literature Prize is awarded by The Swedish Academy [1]. These three institutions also have special Nobel committees that grant a Nobel Prize in the discipline of economics, a prize that was established in 1968 through a grant from Sweden’s central bank, Sveriges Riksbank, to the Nobel Foundation to commemorate the bank’s 300th anniversary [2]. The sixth prize, the Nobel Peace Prize, is awarded by the Norwegian Nobel Committee and selected by a committee organized by the Norwegian Parliament [1].

The history of the Nobel Prize dates back to the Swedish businessman and engineer, Alfred Nobel, who invented dynamite in 1867. This invention was the main reason behind Nobel’s fortune, which was estimated at 31.5 million Swedish Krone [3]. In 1895, Alfred Nobel recommended directing his fortune to the creation of an institute to award prizes to those who contributed significant works that benefited chemistry, physics, physiology or medicine, literature, economics, and/or peace, with each prize named in his honour [4]. Alfred Nobel died in 1896, leaving behind substantial achievements, which have contributed positively to the progress of many areas of science and culture and, through his will, directed most of his fortune to the creation of these prizes. The first prize was awarded in 1901, which coincided with the fifth anniversary of Alfred Noble’s death, and from then on, the Nobel Prize has been awarded annually to any individual or team in the specified disciplines.

World War II (WWII) affected the distribution of Nobel Prizes, which caused the prize to be suspended from 1940 to 1942, and in 1943 the prize was granted only for achievements in medicine, chemistry, and physics [5]. Between 1901 and 1940, Nobel Prize recipients were predominantly from Germany and France. This changed significantly during WWII, largely due to a mass migration that occurred at the time, resulting in a complete collapse of science in Germany and Eastern Europe after World War II. Despite this, and due to the effects of fascist regimes, the impact of migration on science led to an increase in United States recipients receiving a Nobel Prize [6].

Nussbaum [7] indicated the limited number of women pursuing higher education in research as a potential factor contributing to fewer women Nobel laureates. However, Nussbaum also noted that the proportion of women involved in research was much higher in developed countries compared to those in developing countries [7]. Since research is time-consuming, research was considerably more challenging for women, compared to men, due to difficulties in maintaining family-work balances [8]. In the last 30 years, however, this pattern has changed, resulting in more women taking up research as a profession; modern women have become more career-oriented, with increased involvement of women in science over the last few decades [9].

The purpose of this paper is to analyze the awarding of a Nobel Prize while considering the nature of the association between each recipient’s *Country* of nationality, the *Discipline* in which the award was received, the recipient’s *Gender*, and the *Time* period in which the recipient was awarded the prize. This paper extends the study of Alhuzali, Stojanovski, and Beh [10], where the attention was confined to the association between only two of the categorical variables, *Country* and *Discipline*, by using simple correspondence analysis (SCA). This paper will extend their study by illustrating the applicability of multiple correspondence analysis (MCA) to represent the underlying structures in the data by simultaneously examining the association between *Country*, *Discipline*, *Gender*, and *Time*. MCA provides a powerful tool to explore complex Nobel prize data, covering multiple variables including *Country*, *Discipline*, *Gender*, and *Time* Period to demonstrate deeper insights into how the underlying variables are related, with the main benefit being the ability to represent multiple categorical variables visually, which standard statistical tests do not allow.

This paper presents the MCA of the association between the four variables. In Section 2, a brief description of the data is provided (Section 2.1) as well as the specifics of the MCA technique (Section 2.2). Section 3 provides a comprehensive description of the results from the MCA of the data where Section 3.1 gives a general overview of the Nobel Prize data based on the gender of the recipient during three different time periods (1901–1940, 1941–1980, and 1981–2018). An overview of the Nobel Prize data based on the gender of the recipient is provided in Section 3.2, while Section 3.3 discusses the distribution of Nobel Prizes during these three time periods. Section 3.4 provides a detailed assessment of the association between the four variables, *Country*, *Discipline*, *Gender*, and *Time* Period, with concluding statements made in Section 4.

## Methods

### Data

This study examines the awarding of Nobel Prizes from 1901 to 2018 across eight-developed countries. The data examined was obtained from two official websites: the Nobel Foundation [2] and the Encyclopedia Britannica [11]. The study consists of four categorical variables: *Country*, *Discipline*, *Gender*, and *Time*. The *Country* variable comprises eight categories: Canada (CA), France (FR), Germany (DE), Italy (IT), Japan (JA), Russia (RU), British Isles (BI), and the United States of America (US). The categories of *Discipline* are Chemistry (Ch), Economy (Ec), Literature (Li), Physiology and Medicine (Me), Peace (Pc), and Physics (Ph). The *Time* variable has three categories: 1901–1940 (P1), 1941–1980 (P2), and 1981–2018 (P3). The categories of *Gender* are coded as M and F for male and female, respectively. The total sample size of the data includes 785 prizes that were awarded between 1901 and 2018.

### Statistical analysis

MCA is an extension of SCA, where the nature of the association between more than two categorical variables can be visually studied. The key benefit of MCA is that it is a powerful multivariate statistical technique used in large, complex datasets to analyze and graphically represent multivariate categorical data; see, for example, Greenacre [12], Greenacre and Blasius [13], and Beh and Lombardo [14] for a discussion of many issues concerning MCA. While these contributions discuss in great detail various aspects of the classical approach to MCA there is also a wealth of application that demonstrate the practical benefits of the technique. One may consider, for example, Greenacre [15, 16], Greenacre and Pardo [17], Barth [18] and Dungey, Tchatoka and Yanotti [19] which cover the disciplines of medicine, health, gender attitudes and finance. To describe this approach, consider a multiway contingency table, ***N***, formed from the cross-classification of *Q* categorical variables, *X*_1_, *X*_2_,….,*X*_*Q*_ that consist of *J*_1_, *J*_2_,….,*J*_*Q*_ categories respectively. For our multiway contingency table, ***N***, we define the total number of categories as $J=\sum_{q=1}^{Q}J_{q}$.

The first step to applying MCA to a *Q*-way contingency table is to transform it into the form of a two-way matrix. Here we will confine our attention to using crisp coding; one of the most commonly implemented approaches of coding used for MCA. Crisp coding is performed so that either a 0 or 1 is allocated to each individual based on whether a characteristic of a variable is observed (1) or not (0); see, for example, Beh and Lombardo [14, 15]. For our *Q*-way contingency table, the *n*×*J*_*q*_ matrix ***Z***_*q*_ is the indicator matrix for *q*th variable. This is a matrix of 0’s and 1’s; for each row there are *J*_*q*_−1 zeros for categories where the observation was not made, and a single 1 for the observed category. Thus, a super-indicator matrix ***Z*** of size *n*×*J* is formed by concatenating the *Q* indicator matrices such that:

$$Z=[Z_{1}\;Z_{2}\cdots Z_{Q}].$$

Therefore, the row marginal frequencies of ***Z*** are all equal to *Q*, and the column frequencies of ***Z*** are equal to the marginal frequencies of the *Q*-way contingency table. The total of ***Z*** is therefore *nQ*. The matrix ***Z*** will often consist of a large number of rows when the sample is very large. To avoid this problem, an alternative strategy can be used by defining and analyzing the Burt matrix ***C*** [20]. The Burt matrix is of size *J*×*J* and is obtained directly from the indicator matrix ***Z*** such that:

$$C=Z^{T}Z=\left(\begin{array}{cccc} {Z_{1}}^{T}Z_{1} & {Z_{1}}^{T}Z_{2} & \cdots & {Z_{1}}^{T}Z_{Q}\\ {Z_{2}}^{T}Z_{1} & {Z_{2}}^{T}Z_{2} & \cdots & {Z_{2}}^{T}Z_{Q}\\ \vdots & \vdots & \ddots & \vdots \\ {Z_{Q}}^{T}Z_{1} & {Z_{Q}}^{T}Z_{2} & \cdots & {Z_{Q}}^{T}Z_{Q} \end{array}\right)=n\left(\begin{array}{cccc} D_{1} & P_{12} & \cdots & P_{1Q}\\ P_{21} & D_{2} & \cdots & P_{2Q}\\ \vdots & \vdots & \ddots & \vdots \\ P_{Q1} & P_{Q2} & \cdots & D_{Q} \end{array}\right).$$

where, ***P***_12_, for example, is the *I*_1_×*I*_2_ two-way matrix of proportions for the first variable and the second variable, and ***D***_1_ denotes the diagonal matrix of marginal proportions for the first variable. Given the symmetric nature of ***C***, an eigen decomposition can be performed so that:

$${\left(D^{C}\right)}^{-1}(P^{C}-r_{Q}{r_{Q}}^{T}){\left(D^{C}\right)}^{-1}=(U^{C}){D_{\lambda }}^{C}{\left(U^{C}\right)}^{T},$$

where

$$(U^{C}){D_{\lambda }}^{C}{\left(U^{C}\right)}^{T}=I,$$

$$P^{C}=\frac{1}{Q^{2}n}C,$$

$$D^{C}=\frac{1}{n}diag(C).$$

The Pearson chi-squared statistic of the Burt matrix, $X_{C}^{2}$, consists of the sum of the chi-squared statistics for each two-way appearing in the off-diagonal sub-matrices of ***C*** and the diagonal sub-matrices holding its marginal proportions, namely:

$$X_{C}^{2}=\sum_{q=1}^{Q}\sum_{q^{\prime }}^{Q}X_{qq\prime }^{2}$$

$$=\sum_{q\neq q^{\prime }}X_{qq\prime }^{2}+\frac{\left(J-Q\right)}{Q^{2}},$$

where $X_{qq\prime }^{2}$ is the chi-squared statistic for the (*q*, *q*′) off-diagonal sub-matrix of ***C*** [21]. By adopting the terminology commonly used in correspondence analysis, the quantity *X*^2^/*n* is proportional to Pearson’s chi-squared statistic and is referred to as the ’total inertia’ of the contingency table; its components are referred to as ’principal inertias’ that are often expressed as a percentage of the total inertia [22]. Therefore, the total inertia of the Burt matrix is:

$$\frac{X_{C}^{2}}{n}=\frac{1}{n}\sum_{q\neq q^{\prime }}X_{qq\prime }^{2}+\frac{\left(J-Q\right)}{nQ^{2}}.$$

The expression after the plus sign (+) represents the total inertia of the sub-matrices that lie along the diagonal of ***C***. Thus, the total inertia of the Burt matrix is artificially inflated by a factor of (*J*−*Q*)/*nQ*^2^); this inflation can be problematic, particularly if the number of categories is much larger than the number of variables. Since the existence of the off-diagonal sub-matrices of the Burt matrix may affect the magnitude of the total inertia, Greenacre [22] proposed the method of Joint Correspondence Analysis (JCA) by developing an algorithm to solve this issue. His algorithm starts by minimizing

$$SSE=\frac{1}{{nQ}^{2}}trace\;\left[{\left(D^{C}\right)}^{-1}(C-\hat{C}){\left(D^{C}\right)}^{-1}{\left(C-\hat{C}\right)}^{T}\right],$$

where $\hat{C}$ is a low-rank approximation of the Burt matrix ***C***, and

$$\hat{C}=Q^{2}n(r_{Q}){\left(r_{Q}\right)}^{T}+Q^{2}nD^{C}U^{C}{D_{\lambda }}^{C}{\left(U^{C}\right)}^{T}D^{C}+E,$$

where *r*_*Q*_ is the vector of column proportions, (***U***^*C*^)^***T***^***D***(***U***^*C*^) = *Q****I***, and ***E*** is a super-diagonal matrix of “error” terms that are iteratively estimated. Thus, SCA is performed on the modified Burt matrix in order to obtain a new modified Burt matrix by replacing the diagonal blocks with estimates from the new solution. This algorithm is repeated several times until convergence is achieved, and the fit of the off-diagonal blocks is improved with each iteration [23]. Therefore, the total inertia can be expressed in terms of the elements of ***D***_*λ*_^***J***^ as:

$$\frac{X_{J}^{2}}{n}=\frac{1}{n}\sum_{m=1}^{M}{\left(\lambda_{m}^{J}\right)}^{2},$$

where $\lambda_{m}^{J}$ is the *m*th principal inertia (eigen value) where *M* is at most (min(*n*, *J*)−1) and is the maximum number of dimensions needed to visualize the association. Since, for many applications of MCA and JCA, *J*≪*n*, *M* will be at most *J*−1, although Greenacre [22] showed that the optimal number of dimensions needed when analyzing the Burt matrix or performing JCA is *J*−*Q*.

## Results

### Nobel Prize data overview

Table 1 is the four-way contingency table of the categorical variables *Country* and *Discipline*, based on *Gender* and *Time*. By performing a chi-squared test of independence on this table, we find that there is a statistically significant association between the four variables (*X*^2^ = 107.016, *df* = 35, *p*<0.001). Aggregating by *Gender*, there exists a statistically significant association between *Country*, *Discipline*, and *Time* (*p*<0.001). There is also a statistically significant association between *Country*, *Discipline*, and *Gender* (*p*<0.001). From Table 1, it is noted that the majority of the Nobel Prize recipients are male and account for approximately 742 of the 785 prizes (or 94.5%) awarded between 1901 and 2018, with only 43 prizes (or 5.5%) awarded to female recipients. In the following two sections, an exploration of the awarding of the Nobel Prize data will be provided, with a particular focus on the *Gender* of the recipient and the *Time* period during which the award was received.

**Table 1:** Four-way contingency table from the cross-classification of *Country* and *Discipline*, based on *Gender* and *Time* period.

Gender		Discipline						Total
Time period		Ch	Ec	Li	Me	Pc	Ph	
Males 1901–1940	CA	0	0	0	2	0	0	2
	FR	5	0	7	4	0	5	21
	DE	16	0	5	9	3	11	44
	IT	0	0	2	1	1	2	6
	RU	0	0	1	2	0	0	3
	BI	6	0	3	7	5	10	31
	US	3	0	2	4	6	6	21
	Total	30	0	20	29	15	34	128
1941–1980	CA	1	0	1	1	1	0	4
	FR	1	0	5	6	0	2	14
	DE	10	0	2	9	4	9	34
	IT	1	0	2	3	0	1	7
	JP	0	0	1	0	1	3	5
	RU	2	1	3	0	2	7	15
	BI	15	4	3	12	2	11	47
	US	21	9	7	48	8	39	132
	Total	51	14	24	79	18	72	258
1981–2018	CA	5	4	1	3	0	4	17
	FR	7	3	4	2	1	6	23
	DE	4	1	1	8	0	13	27
	IT	0	1	1	1	0	2	5
	JP	7	0	2	5	0	8	22
	RU	0	1	0	0	1	5	7
	BI	10	7	4	16	4	7	48
	US	53	51	2	43	4	54	207
	Total	86	68	15	78	10	99	356
Females 1901–1940	FR	2	0	0	0	7	1	10
	IT	0	0	1	0	0	0	1
	US	0	0	1	0	1	0	2
	Total	2	0	2	0	8	1	13
1941–1980	FR	0	0	0	0	4	0	4
	DE	0	0	1	0	0	1	2
	BI	1	0	0	0	4	0	5
	US	0	0	0	2	1	1	4
	Total	1	0	1	2	9	2	15
1981–2018	CA	0	0	0	0	0	1	1
	FR	0	0	0	1	0	0	1
	DE	0	0	1	0	0	0	1
	IT	0	0	0	1	0	0	1
	BI	0	0	1	0	0	0	1
	US	1	1	1	6	1	0	10
	Total	1	1	3	8	1	1	15

### Overview of Nobel Prize data based on gender of the recipient

Table 1 shows that, when compared with females in other countries, more females in the USA and France received Nobel Prizes, with notably more female recipients from the USA in the more recent time period (1981–2018) while there were more females from France in the earlier time period (1901–1940). Of all female recipients, 37% were from the USA, with the most prominent disciplines among female recipients in the USA in medicine (50%) and peace (19%). Of all female laureates, 35% were from France, with the highest proportion of French female recipients in the peace discipline (73%). While prizes in Peace followed by Medicine were the most prominent among female recipients (42% and 23% respectively), the economics prize was only awarded once to a female (2%), while the remaining prizes among females were distributed evenly between the disciplines of chemistry and physics (9% each), with 14% of female prizes in literature.

While more males than females are awarded Nobel Prizes, female recipients in France received more of the overall peace prizes (18%) than their male counterparts (2%). By comparing the male recipients in the eight countries, there were more Nobel Prize recipients in the USA than in the other seven countries; the award disciplines including chemistry, economics, medicine, peace, and physics, with US recipients receiving 46%, 73%, 51%, 42%, and 48% of these prizes, respectively. However, of the male recipients, those in France dominated the literature Nobel Prize, receiving approximately 27% of them.

### Overview of Nobel Prize data based on time periods

From Table 1, it is observed that recipients from Germany received more Nobel Prizes between 1901 and 1940 than any other country, receiving nearly one third (31%) of the prizes awarded during this period. Most of the prizes among German recipients in this period were in chemistry (36%), physics (25%), and medicine (20%). Over this same time period, France received approximately 22% of the Nobel Prizes awarded. Of the prizes awarded to France over this time period, 23% were in each of chemistry, literature, and peace; while 19%in physics and 13% in medicine. The Nobel Prize was not awarded in economics during this time period. Of the BI prize recipients during this time period, 32% were in physics, while 23% and 19% were awarded Nobel Prizes in medicine and chemistry, respectively.

The BI received 19% of Nobel Prizes between 1941 and 1980; the largest share of the Nobel Prizes after the USA, which received 50% of the awards over this time period. Most of the prizes to BI and American recipients combined were in chemistry (20%), medicine (33%), and physics (27%). The USA continued with its high level of achievement between 1981 and 2018, receiving 58% of Nobel Prizes awarded, with about 15% of prizes awarded to each of chemistry, physics, and medicine disciplines.

Between 1941 and 1980, the Nobel Prize in economics was awarded for the first time, with the USA receiving 64% of the total economic prizes awarded during this period, the highest percentage compared to other countries during this time period. The USA dominated Nobel Prizes during this period, receiving nearly half of the total awards presented; with recipients from the USA being awarded 18% of prizes in medicine, while those in physics and chemistry received 15% and 8%, respectively, of the Nobel Prizes awarded in those disciplines during this period. From 1980 onward, Canada and the USA dominated the economics prize, potentially reflecting their dominance in neoliberal policies that have dominated the world economic system [24]. Economics is very much considered a USA based intellectual discipline, with the international graduate study of economics increasingly following and reflecting the model in the USA [24].

Migration is an important contributing factor to the success of America in the Nobel Prize, particularly in the field of economics from 1980 onwards [25], with immigrants to the United States who won a Nobel Prize ranking second only to United States laureates [25], with their number exceeding the number of laureates born in any country alone. Najam indicated that there are several reasons that America excels in economics, including high immigration rates and world class education along with the openness of American academics to scholars internationally [25].

There appears to be a Russian and Italian dominance of the Nobel Prize for Literature in the first half of the twentieth century. This dominance appears to later diminish due to the then questioning of the value of the Nobel Prize for Literature in Italy, with accusations driven by partisan politics that attracted attention from the award committee, which was speculated as withholding the award from loyalists to dictatorial and fascist regimes, hence potentially detracting from the literary content of nominated works [26]. Furthermore, it was noted in Engdahl’s book, ‘Nobel Sensibility’, that due to the growing interest in the Literature Nobel Prize, and due to its cultural and literary status, awardees have become modern literary legitimacy, triggering a trend that has caused the removal of many great writers of the twentieth century from the list [27].

### Multiple correspondence analysis results

**Fig 1:**
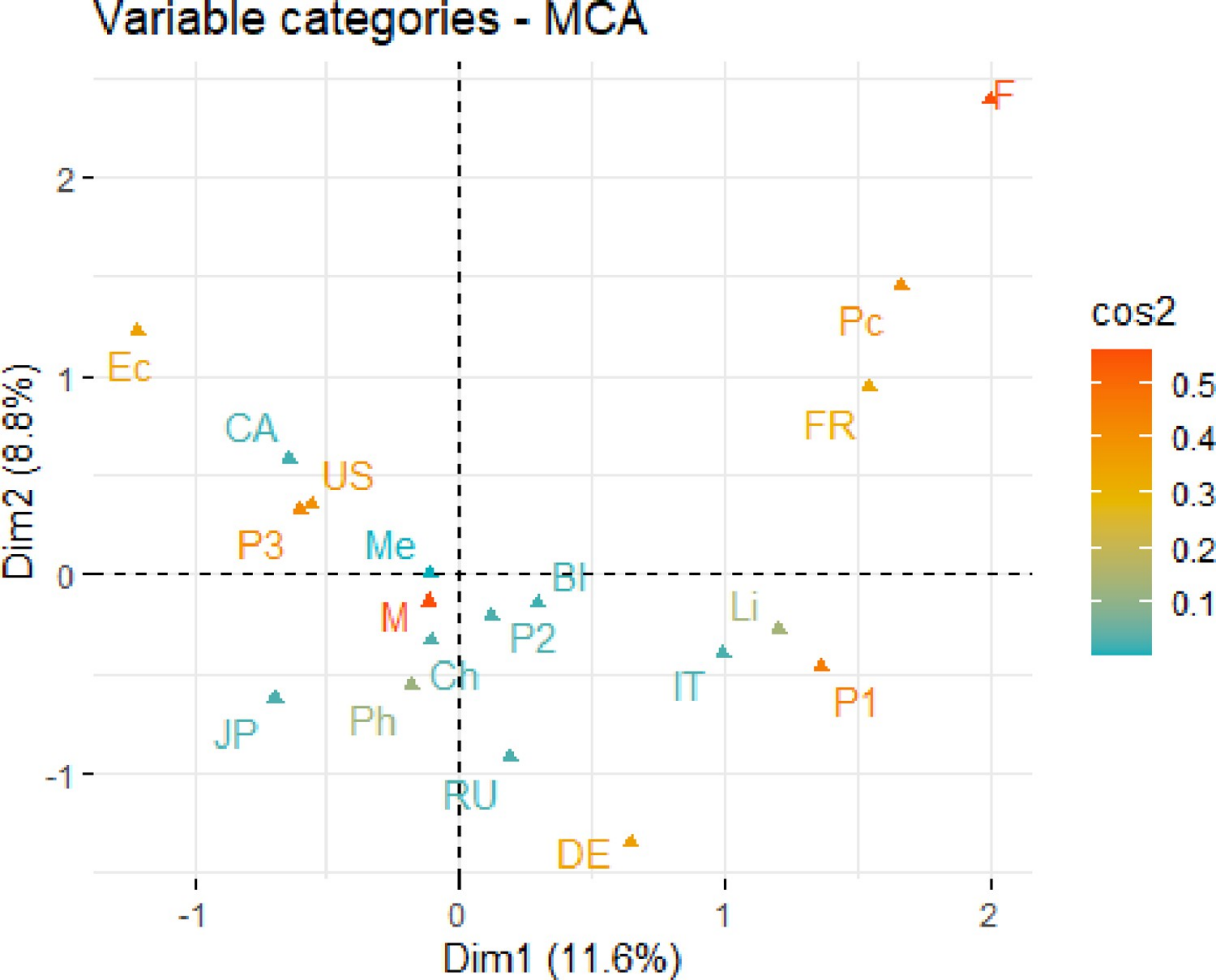
Two-dimensional correspondence plot using MCA.

**Fig 2:**
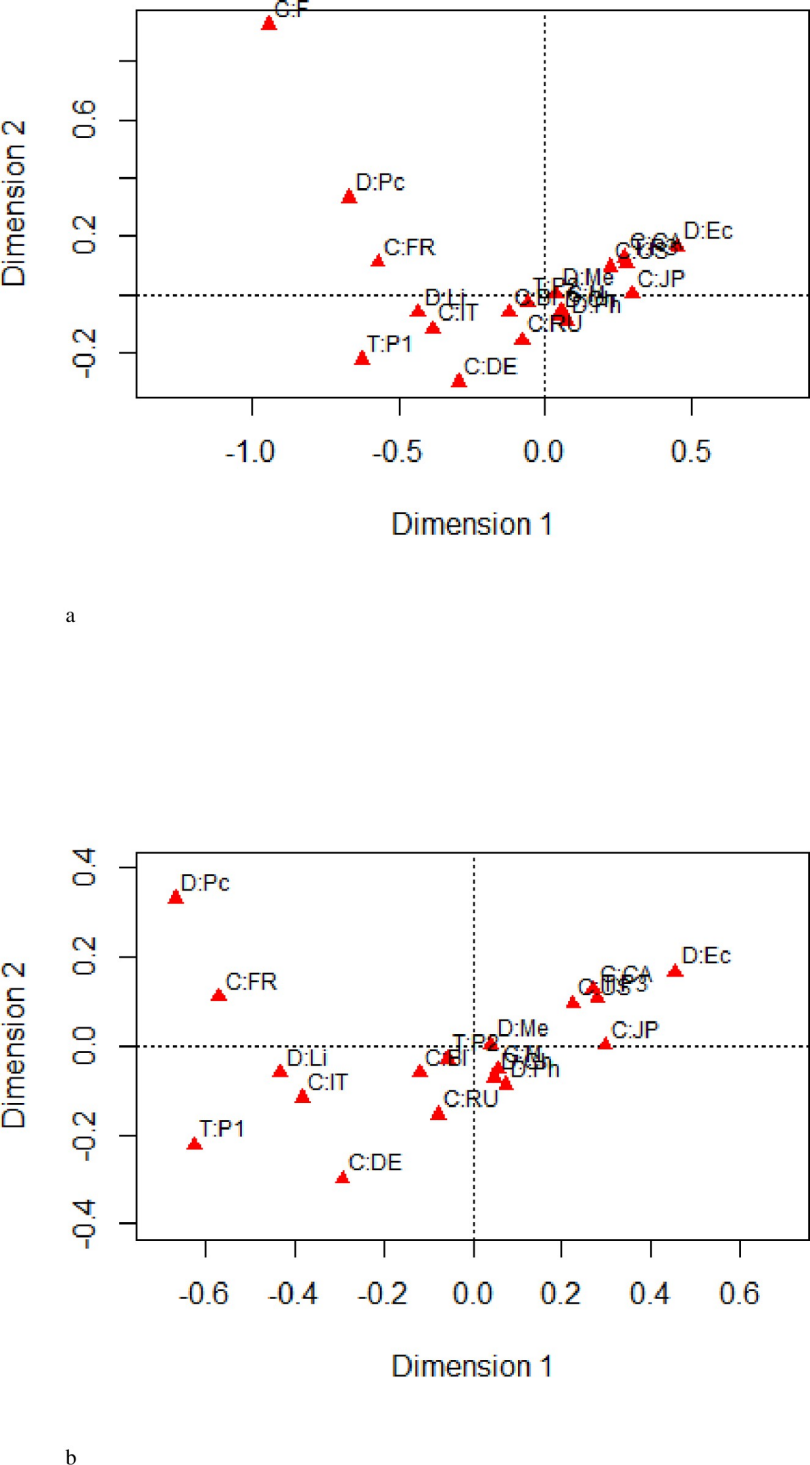
a. Two-dimensional correspondence plot of Table 1 from the JMCA. b. Zoomed in version of Fig 2A after removal of the highlighted point.

While Section 3.1 provides a numerical summary (through simple percentages) of the association between the variables *Country*, *Discipline*, *Gender*, and *Time*, the focus will now shift to the visualization of this association. The data in Table 1 is consequently analyzed using MCA, where the calculations and visual summary are produced using the “ca” package of Nenadić and Greenacre [28]. MCA is performed by first transforming Table 1 into its corresponding Burt matrix, and then performing a simple correspondence analysis (SCA) on this Burt matrix. Fig 1 shows the two-dimensional correspondence plot resulting from the MCA. Since the first two dimensions account for 11.6% and 8.8%, respectively, of the total inertia, Fig 1 visually describes only approximately 20% of the association between the four categorical variables. With such a small percentage, Fig 1 is deemed to be a poor visual summary of the association. To subsequently improve the quality of the two-dimensional display, JCA was performed using the “ca” package. The correspondence plot generated from this analysis is provided in Fig 2 and now visually accounts for 81% of the association between the four categorical variables. The two-dimensional correspondence plot generated from the JCA of the data in Table 1 hence provides a much better visual summary of the association between the four categorical variables. Except for a rotation about the second dimension, the configuration of points in Figs 1 and 2 are relatively similar. Therefore, reducing the impact of the diagonal sub-matrices of the Burt matrix has not substantially altered the configuration of points for the categories of *Country*, *Discipline*, *Gender*, and *Time*, rather, it has greatly improved the quality of the display. The extreme value in the upper left corner of Fig 2A made it difficult to view the associations so the figure with the removal of the extreme value (corresponding to Females) is displayed in Fig 2B.

To provide a more detailed interpretation of the association, consider the following points that are made based on the results summarized in Table 2. The first three columns of Table 2 summarize the percentage of the total inertia contributed by each category and the principal coordinates for the two dimensions, respectively. The correlation (cor) and the contribution (ctr) of each category are displayed in the fourth and fifth columns. From Table 2, the quality for the categories USA, Germany, and France of the *Country* variable are 0.934, 0.898, and 0.863, respectively. Therefore, Fig 2 depicts 93%, 90%, and 86% of the contribution made by the USA, Germany, and France, respectively, that visually depicts the association between *Country*, *Discipline*, *Gender*, and *Time*. While these are the best-represented categories in the correspondence plot, the country with the poorest quality of representation is Russia, in which only 10% of its contributions to the association are depicted in Fig 2; the remaining 90% can be accounted for by the third and higher dimensions.

**Table 2:** Contribution of categorical variables to Fig 2 from joint correspondence analysis.

Columns						
	name	% inertia	Dim. 1 Princ. Coor	Dim. 2 Princ. Coor	cor	Ctr
1	C: Canada	6.1	0.270	0.127	0.625	0.008
2	C: France	6.3	−0.572	0.110	0.863	0.103
3	C: Germany	5.8	−0.294	−0.300	0.898	0.066
4	C: Italy	6.2	−0.385	−0.117	0.442	0.012
5	C: Japan	6.1	0.297	0.001	0.331	0.009
6	C: Russia	6.1	−0.080	−0.156	0.104	0.003
7	C: British Isles	5.2	−0.120	−0.062	0.590	0.008
8	C: USA	3.7	0.225	0.095	0.934	0.079
9	D: Chemistry	4.9	0.047	−0.071	0.318	0.005
10	D: Economics	6.1	0.454	0.167	0.815	0.082
11	D: Literature	6.1	−0.434	−0.062	0.683	0.051
12	D: Medicine	4.7	0.039	0.003	0.091	0.001
13	D: Peace	6.5	−0.669	0.331	0.931	0.131
14	D: Physics	4.7	0.075	−0.088	0.532	0.011
15	G: Male	0.4	0.055	−0.054	0.979	0.009
16	G: Female	6.8	−0.945	0.924	0.979	0.152
17	T: 1901–1940	6.2	−0.626	−0.224	0.953	0.174
18	T:1941–1980	4.2	−0.057	−0.028	0.090	0.003
19	T: 1981–2018	3.9	0.280	0.106	0.874	0.092

From Fig 2, it is clear that female recipients of the Nobel Prizes from France are strongly associated with the Peace prize; a point that was noted in Section 3.2 when summarizing the association using percentages. Nobel laureates from Italy dominated the Nobel Prizes in literature from 1901 to 1940. There is a strong association between recipients from Canada and the USA and the awarding of the Nobel Prize in Economics, particularly during the 1981–2018 time period. It is clear also that there is a strong association from 1941 to 1980, the categories M, BI, Me, Ch, and Ph, since they are tightly clustered together. This implies that men in the BI are strongly associated with being awarded Nobel Prizes in the disciplines of medicine, chemistry, and physics during the period 1941–1980. However, since the proximity of their points lies close to the origin, their contribution to the association is not as dominant as other category combinations. Moreover, the categories RU and JP are associated with the Ph category, indicating that laureates from Russia and Japan are strongly associated with the discipline of physics. As mentioned in Section 3.3, the recipients of Nobel Prizes from the US were dominant from 1981 to 2018, which is also evident in Fig 2, where the position of the USA and the period from 1981 to 2018 are virtually identical. Similarly, the categories BI and the period from 1941 to 1980 are strongly associated due to their tight proximity. This means that the Nobel laureates from the BI dominated the prizes between 1981 and 2018 in a variety of disciplines. Furthermore, DE and IT are the closest categories of the *Country* variable in regards to the *Time* category from 1901 to 1940, which implies that this is the most common period that recipients from Germany and Italy received Nobel Prizes when compared with other periods.

## Discussion

In this paper, MCA was used to graphically represent and interpret the associations among the *Country* of the nominated individual (or of the nominated team), *Discipline* in which the Nobel Prize was awarded, *Time* period that the recipient received the award, and *Gender* of the recipient. Furthermore, JCA was used to improve the quality of the two-dimensional display using the “ca” package. This technique provides a comprehensive description of the association between these variables. This paper also demonstrated the value of MCA for the assessment of temporal data. In this instance, the duration over which Nobel prizes were awarded was investigated. Three time periods were investigated for the present study: 1901–1940, 1941–1980 and 1981–2018, and the relationship of time period with country and discipline was assessed. The MCA enabled the complex multivariate association between these three variables to be assessed simultaneously, demonstrating how MCA can represent, describe and analyse temporal data effectively. The results from the MCA show not only a statistically significant association between the variables *Country*, *Discipline*, *Gender*, and *Time*, but also describe how specific categories are related to each other. However, by the nature of MCA, the variables are assumed to be symmetrically associated; where they are all treated as predictor variables. That stated, one may wonder if, for example, a Nobel laureate is female, what impact her gender has on the discipline in which she was awarded a Nobel Prize or the time period in which the prize was awarded. An analyst may also be interested in examining the impacts on discipline or gender, based on the country a laureate comes from. Therefore, it is important to consider how one may expand the MCA of this data by reflecting upon the asymmetric association structure that may exist (either by design or by the nature of the association being investigated) between categories. Doing so may offer additional insight into the awarding of the Nobel Prize.

## References

[1] Levinovitz AW, Ringertz N. The Nobel Prize: the first 100 years. Singapore: World Scientific Publishing; 2001.

[2] NobelPrize.org. All Nobel Prizes [online]. 2019 [cited 2019 November 25]. Available from: https://www.nobelprize.org/prizes/lists/all-nobel-prizes/.

[3] The Nobel Peace Prize. Alfred Nobel’s Fortune [online]. 2019 [cited 2020 November 25]. Available from: https://www.nobelpeaceprize.org/History/ Alfred-Nobel-s-fortune

[4] Morais FB. Vision and the Nobel Prize. Arq Bras Oftalmol. 2018; 81: 161–165. doi: 10.5935/0004-2749.20180035 29846418

[5] The Nobel Prize. 2020 [cited 2020 November 25]. Available from: https://www.nobelprize.org/.

[6] Weiss E. The Impact of the Intellectual Migration on the United States and Eastern Europe. The Nobel Prize Winners Science. 1998 [cited 22 February 21]. Available from https://www.vanderbilt.edu/AnS/physics/brau/H182/Term%20Papers/Eric%20Weiss.html

[7] Nussbaum MC. Women’s education: a global challenge. Signs J Women Cult Soc. 2004; 29: 325–355.

[8] Emans SJ, Austin SB, Goodman E, Orr DP, Freeman R, Stoff D, et al. Improving adolescent and young adult health—training the next generation of physician scientists in transdisciplinary research. J Adolesc Health. 2010; 46: 100–109. doi: 10.1016/j.jadohealth.2009.10.004 20113915

[9] Modgil S, Gill R, Lakshmi Sharma VL, Velassery S, Anand A. Nobel nominations in science: constraints of the fairer sex. Ann Neurosci. 2018; 25: 63–78. doi: 10.1159/000481906 30140118 PMC6103368

[10] Alhuzali T, Stojanovski E, Beh EJ. Correspondence analysis approach to examine the Nobel Prize. In: Elsawah S, editor. MODSIM2019, 23rd International Congress on Modelling and Simulation. Modelling and Simulation Society of Australia and New Zealand, December 2019; 2019. pp. 284–290.

[11] Bergmann PG. Relativity. In: The new encyclopedia Britannica. Vol. 26. Chicago: Encyclopedia Britannica (2019); 1993. pp. 501–508.

[12] Greenacre MJ. Theory and applications of correspondence analysis. London: Academic Press; 1984.

[13] Greenacre M, Blasius J. Multiple correspondence analysis and related methods. London: Chapman & Hall/CRC Press; 2006.

[14] Beh EJ, Lombardo R. Correspondence analysis: theory, practice, and new strategies. Chichester: Wiley; 2014.

[15] Greenacre M, Correspondence analysis in medical research, Statistical Methods in Medical Research 1992; 1: 97–117. doi: 10.1177/096228029200100106 1341654

[16] Greenacre M. Correspondence analysis of the Spanish National Health Survey, Gaceta Sanitaria 2002; 16: 160–170 doi: 10.1016/s0213-9111(02)71648-8 11958753

[17] Greenacre M, Pardo R. Multiple correspondence analysis of subsets of response categories, In: Greenacre M, Blasius J, editors. Multiple Correspondence Analysis and Related Methods. London: Chapman & Hall/CRC; 2006. p. 197–217.

[18] Barth A. The changing nature of attitude constructs: an application of multiple correspondence analysis on gender role attitudes, Quality & Quantity 2016; 50: 1507–1523.

[19] Dungey M, Tchatoka FD, Yanotti MB. Using multiple correspondence analysis for finance: A tool for assessing financial inclusion, Int Rev Fin Ana. 2018; 59: 212–222.

[20] Burt C. Intelligence and social mobility. Br J Stat Psychol. 1961; 14: 3–24.

[21] Beh EJ, Lombardo R. Multiple and multiway correspondence analysis. Wiley Interdiscip Rev Comput Stat. 2019;11: e1464. doi: 10.1002/wics.1464

[22] Greenacre MJ. Correspondence analysis of multivariate categorical data by weighted least-squares. Biometrika 1988; 75: 457–467.

[23] Kamalja KK, Khangar NV. Multiple correspondence analysis and its applications. Electron J Appl Stat Anal. 2017;10: 432–462.

[24] Lindbeck A. (1999). "The Sveriges Riksbank Prize in Economic Sciences in Memory of Alfred Nobel." Available from nobelprize. Org.

[25] Najam A. (2016), America’s Nobel success is the story of immigrants. The Conversation [Internet]; 2016 [cited 2022 February 21]. Available from https://theconversation.com/americas-nobel-success-is-the-story-of-immigrants-67219

[26] Mallozzi I. (2016). Six Italian Nobel Prize Winners in Search of a National Identity; 2016 [cited 2022 February 21]. Available from https://theculturetrip.com/europe/italy/articles/six-italian-nobel-prize-winners-in-search-of-a-national-identity/

[27] Engdahl H. (2010). A Nobel sensibility. World Policy Journal 2010; 27(3): 41–45.

[28] Nenadić O, Greenacre M. Correspondence analysis in R, with two- and three-dimensional graphics: the ca package. J Stat Softw. 2007; 20(3): 13 pages.

